# Etymologia: *Mycobacterium chimaera*

**DOI:** 10.3201/eid2303.ET2303

**Published:** 2017-03

**Authors:** Ronnie Henry

**Keywords:** etymologia, Mycobacterium chimaera, bacteria, tuberculosis and other mycobacteria, opportunistic pathogen

## *Mycobacterium chimaera* [miʺko-bak-tērʹe-əm ki-mērʹə]

Formerly an unnamed *Mycobacterium* (Figure) sequevar within the *M. avium*–*M. intracellulare*–*M. scrofulaceum* group (MAIS), *M. chimaera* is an emerging opportunistic pathogen that can cause infections of heart valve prostheses, vascular grafts, and disseminated infections after open-heart surgery. Heater–cooler units used to regulate blood temperature during cardiopulmonary bypass have been implicated, although most isolates are respiratory. In 2004, Tortoli et al. proposed the name *M. chimaera* for strains that a reverse hybridization–based line probe assay suggested belonged to MAIS but were different from *M. avium*, *M. intracellulare*, or *M. scrofulaceum*. The new species name comes from the chimera, a mythological being made up of parts of 3 different animals.

**Figure:**
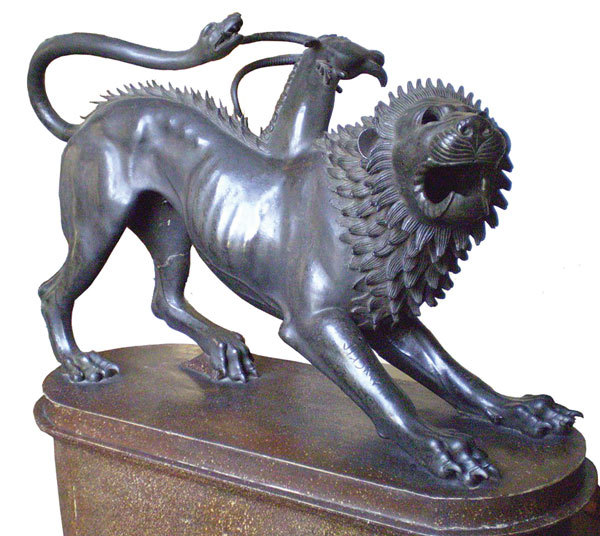
Etruscan bronze statue depicting the legendary monster, the Chimera. National Archaeological Museum, Florence.  Photograph by Lucarelli (Wikimedia Commons)
